# Ecological and Evolutionary Implications of Microbial Dispersal

**DOI:** 10.3389/fmicb.2022.855859

**Published:** 2022-04-06

**Authors:** Gordon F. Custer, Luana Bresciani, Francisco Dini-Andreote

**Affiliations:** Department of Plant Science and Huck Institutes of the Life Sciences, The Pennsylvania State University, University Park, PA, United States

**Keywords:** coalescence, community assembly, invasion, metacommunity ecology, microbiome

## Abstract

Dispersal is simply defined as the movement of species across space and time. Despite this terse definition, dispersal is an essential process with direct ecological and evolutionary implications that modulate community assembly and turnover. Seminal ecological studies have shown that environmental context (e.g., local edaphic properties, resident community), dispersal timing and frequency, and species traits, collectively account for patterns of species distribution resulting in either their persistence or unsuccessful establishment within local communities. Despite the key importance of this process, relatively little is known about how dispersal operates in microbiomes across divergent systems and community types. Here, we discuss parallels of macro- and micro-organismal ecology with a focus on idiosyncrasies that may lead to novel mechanisms by which dispersal affects the structure and function of microbiomes. Within the context of ecological implications, we revise the importance of short- and long-distance microbial dispersal through active and passive mechanisms, species traits, and community coalescence, and how these align with recent advances in metacommunity theory. Conversely, we enumerate how microbial dispersal can affect diversification rates of species by promoting gene influxes within local communities and/or shifting genes and allele frequencies *via* migration or *de novo* changes (e.g., horizontal gene transfer). Finally, we synthesize how observed microbial assemblages are the dynamic outcome of both successful and unsuccessful dispersal events of taxa and discuss these concepts in line with the literature, thus enabling a richer appreciation of this process in microbiome research.

## Introduction

Biogeographic patterns of microbial communities and community responses to biotic/abiotic stressors have been studied primarily by considering environmental filtering or variance partitioning (i.e., deterministic selection). This is because microbes are presumed to have high dispersal rates, large population sizes, fast growth rates, and a propensity for dormancy (Xu et al., 2020). As such, these aspects collectively corroborate the notion that *“Everything is everywhere, but the environment selects”* (Baas-Becking, 1934). Though the “everything is everywhere” hypothesis was widely accepted through much of the twentieth century, mounting evidence that integrates ecological theory with characterizations of microbial communities has provided support to the contrary and suggests that microbes are instead dispersal-limited (Hubbell, 2001; Vellend, 2016). As such, apart from environmental selection, other community assembly processes, like dispersal, are gaining traction as important drivers of microbial community assembly and turnover (Nemergut et al., 2013; Dini-Andreote et al., 2015; Burns et al., 2017). In this review, we discuss the role of dispersal in micro-organismal ecology and focus on idiosyncrasies between micro and macro systems that may lead to differences in how this fundamental process affects the structure and function of microbiomes. Within the context of ecological implications, we discuss the importance of short- and long-distance microbial dispersal through active and passive mechanisms, the value and concepts associated with species traits that favor dispersal and align these with metacommunity theory. In addition, we conceptualize the generalities of community coalescence (i.e., dispersal of entire communities), a phenomenon largely common in microbial communities. Conversely, we further detail how microbial dispersal can lead to changes in diversification rates dynamically affecting microbial evolution and eco-evolutionary dynamics.

## Defining Microbial Dispersal

Dispersal can be simply defined as the movement of organisms across space and time (Vellend, 2010). However, the process and underlying factors associated with dispersal are much more complex than this definition implies. Dispersal along with drift, selection, and differentiation (i.e., speciation), have conceptually been coined as four fundamental ecological processes responsible for the generation and maintenance of community structure (Vellend, 2010). Dispersal is yet an understudied process that can dynamically affect microbial communities. This general lack of explicit consideration of dispersal as a fundamental mechanism modulating microbial systems is mostly due to challenges associated with quantitative assessments of dispersal in observational microbiome studies, albeit relatively easy to manipulate and study it experimentally. Besides, unlike the other assembly processes, dispersal is not entirely a deterministic or stochastic process (Nemergut et al., 2013; Zhou and Ning, 2017). For example, dispersal may be more of a deterministic process when traits, like spore formation and dormancy, are common to select groups of microbes making them better adept for dispersal. On the other hand, dispersal is more of a stochastic process when density dependence favors more abundant taxa to disperse, as may be the case *via* passive dispersal mechanisms (Nemergut et al., 2013; Zhou and Ning, 2017).

When considering the definition, it is worth noting that the term dispersal does not imply a condition of successful establishment. Terms like “effective dispersal” (Nathan, 2013) or “migration” (Nemergut et al., 2013) can be used when explicitly referring to successful establishment following the dispersal event. In addition, studying the importance of dispersal can be even more complicated by the fact that “unsuccessful” dispersal events (i.e., the movement of species from one location to another followed by short-term persistence) can result in ecologically meaningful outcomes. For example, unsuccessful microbial dispersal has been shown to result in permanent shifts in microbial assemblages with long-lasting impacts on niche structure (Mallon et al., 2018; Amor et al., 2020). Also, it has been shown that transient invaders (or dispersal with fleeting establishment) can induce shifts in microbial communities between alternative stable states (Amor et al., 2020).

### Active and Passive Dispersal in Microbes

**FIGURE 1:**
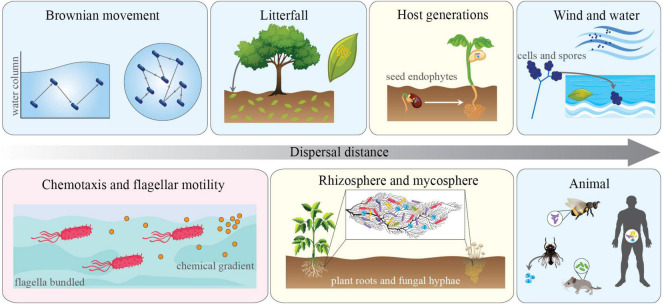
Types and mechanisms of microbial dispersal and the modes by which they operate. Panels depict multiple instances of passive (light blue), active (light red) dispersal. Light yellow panels indicated that both modes of action operate simultaneously. Dispersal range of μM to upwards of hundreds of kilometers is indicated by the horizontal arrow. The figure includes different habitat types and local hotspots that align with the type and selection of traits associated with dispersal in different contexts (see main text).

Dispersal can be conceptually divided into two different mechanisms by which organisms move in space: active and passive (Figure 1). Active dispersal is initiated by inner processes in the microbial cell to direct movement, taking on different forms including hyphal growth and flagellar or ciliary motility. This type of movement can occur in response to predatory pressure, resource availability, or environmental chemical signaling (e.g., chemotaxis) (Hedlund et al., 1991; Fomina et al., 2000; Matz and Jürgens, 2005; McGonigle, 2007). The active movement of microbes is generally assumed to be of greater importance in more diffusible systems (e.g., aquatic habitats). For example, in ocean waters, in the absence of a chemosensory motile trait, bacterial cells rely on Brownian motion to move through the water column. These non-motile bacteria are limited to exploring ∼80 nanoliters per day, while motile bacteria can explore up to 1 ml (Stocker, 2012). Motility has obvious advantages and can facilitate resource capture over patches much larger than that of their non-motile counterparts. As such, traits associated with motility are widespread throughout aquatic microbes and can occur in upwards of 80% of the total community (Blackburn et al., 1998; Fenchel, 2002; Stocker and Seymour, 2012). Worth mentioning, such a type of motility is also important in terrestrial systems. For instance, assisting the movement of species within diffusible hotspots in the soil [e.g., plant-root surfaces (Aroney et al., 2021), fungal hyphae (Pion et al., 2013), see below].

Conversely, passive dispersal occurs when microbial movement is mediated by outside forces, such as the movement of water, wind, soils, animals, or even other microbes (Griffin, 2007; Ingham et al., 2011). This type of dispersal, particularly in terrestrial ecosystems, is often responsible for long-distance movements of microbial cells. For example, it has been shown that pathogenic, prokaryotic microorganisms residing in soils are passively dispersed inter-continentally during dust storms (Griffin, 2007; Gonzalez-Martin et al., 2014). This implies that unlike inter-continental dispersal of macro-organisms, which can take hundreds or thousands of years, global dispersal of microbes through passive mechanisms can occur very rapidly, as recently demonstrated by the shift endemicity to global ubiquity of the SARS-COV-2 virus (COVID-19) over a period of a few months.

### Propagule Pressure

**FIGURE 2:**
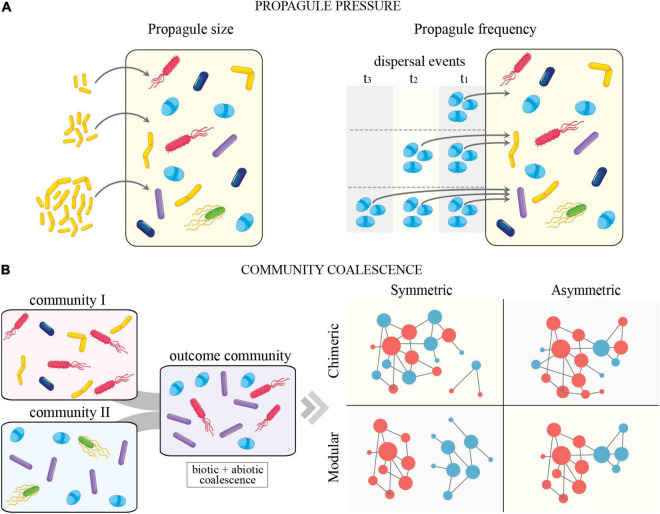
**(A)** Conceptual depiction of propagule pressure (propagule size (left) and frequency (right) in microbial systems. **(B)** Conceptual diagram of community coalescence. Community coalescence is defined as the mixing of entire communities and their environments (*sensu* Rillig et al., 2015, 2016). **(B)** (Left) illustrates community coalescence in microbial systems. **(B)** (Right) depicts a modified version of possible coalescence outcomes from Castledine et al. (2020). In brief, symmetric and asymmetric coalescence indicates the degree to which one input community dominates over another after coalescence. Modular and chimeric coalescence relates to the degree of pre-existing (in previous communities) and novel species interactions (after coalescence), respectively. See Castledine et al. (2020) for additional details.

The concept of propagule pressure depicts the total effort of an organismal introduction in a system (Figure 2). This has been shown to have major importance in explaining the time-dependent success of invasion or dispersal (Lockwood et al., 2005; Simberloff, 2009; Jeschke and Starzer, 2018). Consisting of both frequency (number of dispersal events over time) and size (number of individuals per dispersal event), propagule pressure can vary temporally and have effects on community dynamics. Recently, Albright et al. (2021) provided a synthesis of strategies to prospectively engineer microbiomes based on dispersal dose (i.e., propagule size) and frequency. Within their framework, propagule size is conceptualized to interact with environmental stochastic extinction and density-dependent competitiveness, while dispersal frequency acts to make niche pressure ephemeral and even promote biotic disturbance when taxa are added at different time points. As a general rule, high propagule pressure (i.e., more individuals introduced during a single event and/or more frequent introductions) typically results in a higher probability of establishment (Colautti et al., 2006). However, the importance of the individual components of propagule pressure may be dependent upon the identity of the invader and the composition of the recipient community. In freshwater bacterial mesocosms, competition has been shown to increase the importance of propagule size, while propagule frequency was shown to be most important in communities with lower growth rates (Jones et al., 2017). Other examples with implication for remediation success in soils show that increased propagule pressure results in higher efficiency of removal of environmental contaminants (e.g., petroleum pollution) (Avdalović et al., 2016; Li et al., 2016). Given that diverse microbes can produce an enormous number of propagules and often disperse inter-continentally, some have even gone as far as to describe microbial dispersal as a “microbial conveyor belt” (Mestre and Höfer, 2021). This concept suggests that microbes move around the globe through recurrent and cyclic dispersion, allowing selection to act upon specific traits associated with dispersal. Although this is the case for some microbial taxa and/or in particular systems, the explicit consideration of dispersal and propagule pressures at local and regional scales is likely to better inform on the importance of these processes modulating community assembly.

## Microbial Traits and Dispersal

Evolutionary trade-offs mediated by differences in species traits are important for understanding the balance of fundamental processes giving rise to unique ecological assemblages (de Oliveira et al., 2020; Thompson et al., 2020). That is, species traits help to explain the nature of species interactions with the biotic and abiotic edaphic components. Hence, it is possible to assume that depicting specific microbial traits in line with habitat context can lead to a better understanding of how dispersal operates and can be “selected” for across divergent systems. In this section, we consider a set of specific microbial traits associated with microbial dispersal and link these with their relative importance across different environmental contexts. Additionally, we present a short summary of a recent effort made toward the integration of an ecological framework in microbial systems, with a particular focus on trade-offs in traits affecting organismal fitness (Box 1).

**Box 1. Extending Grime’s competitor-stress tolerator-ruderal (C-S-R) framework to microbial traits.**

Early work in macro-organismal systems focused on trade-offs in key fitness traits associated with resource acquisition and efficiency. This led to the development of the *r* vs. *k* framework, the colonization-competition trade-off, and the analogous copiotroph-oligotroph framework for microbes. Conversely, the Competitor-Stress Tolerant-Ruderal (C-S-R) triangle was developed to more holistically explain tradeoffs in life history traits of plants and incorporates the process dispersal (Grime, 1979; Koch, 2001). Recently, the C-S-R framework has been revisited and modified to categorize microbial life histories into three groups that are analogous to the original framework: high yield (Y), resource acquisition (A), and stress tolerance (S), forming the Y-A-S triangle. The Y-A-S framework has provided advances and insights into microbial life history strategies and may serve to further our understanding of traits that facilitate microbial persistence and survival (Wood et al., 2018; Malik et al., 2020). Within the context of the Y-A-S framework, dormancy may link characteristics of the “Y” and “S” lifestyles. Most apparent, dormancy facilitates the persistence of stress tolerant organisms, or “S” lifestyle adapted. The “S” individuals are either capable of dedicating resources to promote stress tolerance or enter a dormancy state depending on the type and intensity of environmental harshness. In this scenario, microbes capable of entering dormancy and disperse long distances may act as colonizers of new habitats. These organisms are likely to reanimate in an environment with excess resources, possibly at early successional stages. In this case, the “Y” life history would then be favored, thus aligning stress tolerators with high yield organisms. In fact, the “Y” and “S” lifestyles likely covary in a negative way with the type “A” organisms (resource acquisition) due to colonization-competition tradeoffs (Tilman, 1994).

### Motility and Chemotaxis

Short-distance active movement by microbes is determined by the presence of traits that allow the organism to sense environmental stimuli (e.g., molecular sensing of chemical gradients or predator pressure) and move toward more favorable conditions (Jarrell and McBride, 2008). These traits include those associated with chemotaxis, flagellar/ciliate motility, and even the “walking legs” of *Mycoplasma* (Miyata, 2008). The importance of motility traits, and thus their prevalence, likely relies upon the diffusibility of the system. For instance, drier soil systems may be less diffuse than wet soils or aquatic systems and thus favor other traits (e.g., antibiotic production/resistance) over the ability to actively move across space (Dini-Andreote et al., 2018). While evolutionary trade-offs likely decreased the prevalence of motility traits in soil, roots or fungal hyphae can act as highways to favor such strategies, thus facilitating the active microbial movement though a heterogenous soil matrix (Pion et al., 2013). On the other hand, in diffuse systems like aquatic habitats or wet soils, motility allows organisms to move across a larger area of the continuous landscape, with direct implications for their ability to exploit nutrient-rich patches (see the “active dispersal” section above for additional detail).

### Spore Formation

While motility allows for short-distance movement, the ability to form spores facilitates the passive dispersal of microbes throughout space and time. Sporulation allows organisms to essentially “pack-up and move-on” to potentially more hospitable conditions. Such a trait can even allow an organism to persist over millions of years (Cano and Borucki, 1995), thus nicely connecting distinct evolutionary time-scales at local communities. The ability to form viable and long-lived spores make possible integral parts of the life cycle for many microbes and can provide resistance to environmental stressors like drought, heat, and even UV radiation (Huang and Hull, 2017). For example, in the pathogenic bacteria *Clostridium difficile*, endospore formation is required to reach the colon of their human hosts (Paredes-Sabja et al., 2014), while in fungi, both sexual and asexual spores can contribute to spatiotemporal dispersal through their often complex and dynamic life cycles (Wyatt et al., 2013).

### Dormancy

Dormancy allows some microbes to enter a reversible state of low metabolic activity when exposed to unfavorable environmental conditions. This promotes the long-term persistence of microbial taxa within a local community (Lennon and Jones, 2011), and assists with long-distance dispersal of taxa *via* passive dispersal. Collectively, it has been shown that such traits are directly associated with patterns of population dynamics *via* temporal dispersal and the storage effect (Warner and Chesson, 1985). The strategy of dormancy can be found in several groups of microbes and encompasses a variety of phenotypes, ranging from latency in viral lifeforms to anhydrobiosis and cryobiosis in tardigrades (Lennon and Jones, 2011; Bertolani et al., 2019). Entering a state of dormancy allows an organism to bypass periods of suboptimal environmental conditions and reanimate when conditions become more favorable. Examples of dormancy in macro-organisms are also common (e.g., seasonal hibernation or extended diapause), though the temporal scale of dormancy in animals and plants is much shorter. It is worth noting that dormancy is also a metabolic costly trait. The mechanisms required for entering and exiting a state of dormancy demands a relatively large energy input, and during periods of dormancy, organisms must maintain cellular repair mechanisms or risk the chance of fatal damage to cellular machinery and loss of the ability to metabolic awaken upon appropriate environmental stimuli (Lennon and Jones, 2011).

Theory from macro-organismal ecology predicts that dispersal and dormancy will negatively covary due to life history trade-offs (Rees, 1993). That is, organisms capable of entering dormant states are not as reliant on changing physical locations, and vice-versa. However, negative covariation between dispersal and dormancy may not always be the case (Buoro and Carlson, 2014), and the opposite relationship may arise when there is a genetic link between traits that affect dormancy and dispersal (Peiman and Robinson, 2017; Wisnoski et al., 2019). In fact, it is plausible to assume that this is the norm rather than the exception for microbes, as dispersal across large spatial scales is largely mediated by passive events and requires dormancy and/or sporulation for long-term cell persistence. Depicting this relationship, Wisnoski et al. (2019) reviewed a set of studies that utilized aquatic zooplanktons as a model to understand positive dormancy-dispersal covariation. Collectively, these studies showed that organisms with durable propagules made a long-lasting contribution to the seed bank and were capable of dispersing longer distances due to their ability to survive the avian digestive tracts (i.e., suboptimal conditions) (Figuerola and Green, 2002; Viana et al., 2016). Thus, microbial reliance upon passive dispersal can result in positive dispersal-dormancy covariation, as traits that facilitate dormancy have an obvious mechanism to affect dispersal and vice-versa.

## Dispersal of Entire Communities and Community Coalescence

In classical ecology, dispersal refers to the movement of single species across space (Vellend, 2010). However, in striking contrast to macro-organismal systems, it is not uncommon for entire microbial communities to be dispersed simultaneously due to their small size and cohabitation of easily moveable units (e.g., soil particles, plant root segments, or animal intestinal tracts) (Figure 2). For an analogy, imagine an entire African savannah community, complete with herbivores, predators, and prey, simultaneously transported to a new location. Upon reaching a new destination, this community (termed as “donor”), begins to interact with the existing ecological system (termed as “resident”) to produce a novel coalesced community. While we know this to be rare in macro-organismal systems, the movement of entire microbial assemblages is likely the norm rather than the exception (e.g., flooding events, soil tillage, animal excretion, shaking hands, etc.) (Rillig et al., 2015, 2016). Through the process known as “community coalescence,” or mixing of complete communities and their environments, microbial assemblages with distinct ecologies and evolutionary histories are combined to produce a variety of outcomes that are dependent upon the type and strength of microbial interactions taking place within each assemblage (e.g., the complexity of trophic and ecological interactions) and their abiotic similarity (Rillig et al., 2015; Castledine et al., 2020).

Community coalescence has been broadly used to manage and manipulate microbiomes in agriculture and clinical settings, even though the concept was only recently formalized (Rillig et al., 2015, 2016). For example, fecal microbiome transplants have been implemented to treat several human diseases, including conditions like recurrent *Clostridium difficile* infection and Chron’s disease (Gupta et al., 2016; Wilson et al., 2019; Sokol et al., 2020). However, desirable outcomes were not consistently achieved, and most of the variation in clinical outcome might relate to our yet inability to properly understand and predict the fundamental basis and outcomes of community coalescence. Likewise, coalescence has also been used to engineer soil microbial assemblages capable of steering restoration in ex-arable lands (Wubs et al., 2016), boosting crop production in organic agriculture (Ramos et al., 2019), or enhancing biomethane production in anaerobic digesters (Sierocinski et al., 2017), and has even been used to reduce nutrient flow-through and enhance recovery at wastewater treatment facilities (Wagner et al., 2002; Priya et al., 2021). Recently, the concept of *meta-gut* was proposed as a conceptual framework that utilizes coalescence to integrate the gut microbiome of excretion with the function these fecal microbiomes provide outside the host. To illustrate this new concept, a study investigated how the fecal microbiome of wild hippos dynamically alters the local biogeochemical processes of the pools these animals inhabit (Dutton et al., 2021).

## Ecological Implications of Microbial Dispersal

Dispersal is a central mechanism in ecology that can only be fully explored in the context of metacommunity theory, i.e., a set of local communities that are linked by dispersal of many potentially interacting species (Leibold et al., 2004). In line with that, all pre-conceptualized paradigms of metacommunities [i.e., patch-dynamic (PD), species-sorting (SS), and mass-effects (ME); in addition to the neutral model (NM)], are actually four different perspectives that characterize how environmental heterogeneity (deterministic selection), and dispersal rates interplay to influence community diversity. In this section, we discuss recent advances in community ecology with a focus on the ecological implications of dispersal on structuring microbial community assemblages in line with metacommunity theory.

### Metacommunity Theory for Microbes

**FIGURE 3:**
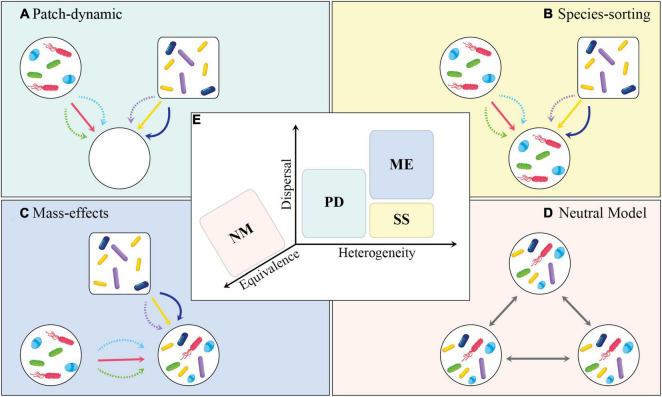
Conceptual diagram depicting the importance of dispersal in metacommunity theory. Each panel shows one of the metacommunity paradigms: **(A)** Patch-dynamic (PD), **(B)** species-sorting (SS), **(C)** mass-effects (ME), and **(D)** neutral model (NM). **(E)** Metacommunity paradigms in relation to their place along the axes of dispersal, environmental heterogeneity, and equivalence. In **(A–D)**, the shapes of communities (i.e., circle and rectangle) indicate differences in abiotic conditions. Different microbial taxa are shown to disperse at different rates, i.e., solid lines indicate higher dispersal rates and dashed lines indicate lower dispersal rates. **(A)** Open patches offer new colonization opportunities as well as evolutionary tradeoffs in colonization and competition. **(B)** Local selection counterbalances the effect of dispersal in determining the community structure. **(C)** High levels of dispersal override the potential effect of local selection. **(D)** Neutral model assumes that all taxa are equally adept at dispersing and the environmental conditions are equivalent across patches. **(A–D)** Are adopted and modified from Leibold et al. (2004), and **(E)** was adopted from Logue et al. (2011).

A metacommunity is defined as a set of local communities with singular composition and functionality linked by dispersal events. Conceptually, theoretical and applied studies have based the metacommunity view through four perspectives: patch-dynamic, species-sorting, mass-effects, and neutral model (Leibold et al., 2004; Figure 3). In short, PD assumes that low levels of dispersal and species trade-off can lead to species coexistence at a regional scale. This implies the existence of distinct time scales between local and regional colonization-extinction dynamics. The SS perspective mostly focuses on the outcomes of species interactions occurring within local communities (habitat patches) that differ in their abiotic and biotic properties. This nicely aligns with the traditional view in microbial ecology (or the niche-based paradigm) and how microbiomes have mostly been studied in terms of variance partitioning. Conversely, ME aligns with SS but in a scenario where the rates of dispersal are too high and can lead to an overall homogenization of local communities over a regional scale. Lastly, NM assumes that species traits and environmental heterogeneity are equivalent across species and habitat types, and, as such, neutral for determining community composition and structure (Perkin et al., 2021).

These four metacommunity paradigms, driven by the movement of individuals among patches, combine ecological processes at local and regional spatial scales (Brown et al., 2017). Hence, metacommunity can be expressed through a set of continuous processes operating within and among interconnected communities. Local processes are mainly based on species interactions, environmental heterogeneity, and *in situ* perturbations, while regional processes are broadly driven by dispersal (Brown and Barney, 2021). Research efforts from macro-organismal ecology have provided examples and insights into the importance of dispersal and habitat heterogeneity for the generation and maintenance of species biodiversity across multiple spatio-temporal scales, including their interactive effects on community α-, β-, and γ-diversities. These can be extended to microbial systems by integrating metacommunity ecology with microbiome research, though these efforts may not be trivial.

### Dispersal and Community Diversity

The rate and magnitude of dispersal have been shown to affect biodiversity at different scales. Predictive models show that intermediate rates of dispersal often result in higher levels of α-diversity (Mouquet and Loreau, 2002). While low rates of dispersal can lead to an overall dominance of highly competitive taxa and augment the importance of ecological drift (i.e., random birth and death events leading to rare species extinction). High levels of dispersal, however, can result in a homogenization of the local and regional species pools. This can lead to an overall short-term increase in stochasticity followed by an increase in deterministic selection at a regional scale that decreases species diversity (Mouquet and Loreau, 2003). As such, it is plausible that intermediate dispersal rates promote the continuous (re-) introduction of taxa and balance the effects of local selection by dispersal from the regional species pool. Additionally, different rates of dispersal across local communities often favor habitat heterogeneity and the differential performance of species across distinct local patches, which collectively account for species coexistence and promote biodiversity in heterogeneous landscapes (Thompson et al., 2020).

Experimental studies in macro- and micro-organism systems and meta-analyses have provided evidence to suggest idiosyncratic outcomes of the relationship between dispersal and local species diversity. Cadotte (2006) reported that while intermediate levels of dispersal can maximize α-diversity in animal communities, such effect does not hold for plant communities. These authors suggested an overall positive relationship, albeit the specific correlation may be non-linear and differ across organismal types. Conversely, Grainger and Gilbert (2016) further reported that approximately 50% of their surveyed experiments found a positive effect of dispersal on α-diversity, of which only 10% found this relationship to display a predicted hump-shaped pattern. In sum, these meta-analyses concluded that divergences between predicted theoretical outcomes and empirical studies are likely the result of three shortcomings in experimental design, including (*i*) failure to incorporate inter-specific differences in dispersal capabilities, (*ii*) heterogeneity in initial community structure and diversity across systems, and (*iii*) the lack of consideration of network structure among patches. All of which offers opportunities to further develop this synthesis in landscape microbial systems and/or *via* the design of prospective experimentation.

In microbial communities, manipulative experiments have provided valuable evidence for the influence of dispersal on local community diversity. Albright and Martiny (2018) examined the influence of dispersal rates in bacterial decomposer communities and showed that dispersal has a rate-dependent effect on community diversity affecting the decomposition rate. It was shown that selection in litter can decrease community diversity in the absence of dispersal due to competitive exclusion combined with ecological drift. Moreover, in another study, it was shown that increasing rates of dispersal result in a hump-shaped pattern of local species diversity (Evans et al., 2017). This pattern occurred because, after a certain level, high dispersal rates weaken local selection. As a follow-up, the authors suggested the strength of local environmental selection to play a role in the extent to which selection and dispersal interact in the system. For instance, by showing that weak selection imposed by low lignin:N ratio related to more labile litter chemistry (Evans et al., 2017).

The effect of dispersal rates on community divergences (i.e., β-diversity) is expected to negatively covary. That is, higher rates of dispersal will result in lower β-diversity by enhancing similarities across local communities (Loreau, 2000; Mouquet and Loreau, 2003; Cadotte, 2006; Grainger and Gilbert, 2016). While this relationship makes intuitive sense and has been supported by literature (Grainger and Gilbert, 2016; Catano et al., 2017), recent experimentation using microbial communities has challenged this notion. Vannette and Fukami (2017) experimentally controlled dispersal vectors to study nectar microbial communities in *Mimulus aurantiacus* plants. These authors reported that nectar communities from the dispersal limited treatments resulted in the lowest measures of β-diversity from the treatment centroid; and the opposite pattern was found in treatments with the highest dispersal. The authors hypothesized that priority effects (i.e., the order of species arrival and their influence on the subsequent community outcome) and competitive interactions between introduced species can significantly alter the magnitude and directionality of the dispersal-diversity relationship. While additional evidence is required to support their findings across divergent systems, these results underscored the importance of incorporating the timing of dispersal as a potential mechanism responsible for divergent outcomes of diversity-dispersal relationships.

### Dispersal and Community Assembly

Community assembly is highly influenced by the timing and frequency of species arrival *via* dispersal and subsequent changes in local abiotic conditions. At the initial phase of community assembly (or primary succession) higher stochasticity in species arrival can be expected (Dini-Andreote et al., 2015). For example, Jackson et al. (2001) observed that during the early stages of drinking water biofilm formation, communities were characterized by different populations that initially colonize the system. Over time, these initial colonizing species can change the local environment and progressively increase the level of environmental selection. As such, the order and timing of species immigration and changes to abiotic conditions, both early or later during community turnover, can affect species distributions and abundances. This concept, termed “priority effects” (Fukami, 2015), refers to the biotic component of historical contingencies and can be explained by two distinct mechanisms triggered by early arriving species: niche preemption and niche modification. Both of which exert continuous influence on community assembly and the subsequent patterns of community dynamics.

In microbial systems, studies examining priority effects have provided insights into the importance of timing of species arrival determining patterns of community assembly and functioning. For example, using wood-decaying fungi as a model system, immigration history was shown to result in large (ca. threefold) differences in fungal species richness and composition, both of which were associated with a similar magnitude in the rate of decomposition and carbon release from wood (Fukami et al., 2010). In another example, Cheong et al. (2021) examined priority effects in polymicrobial biofilms using a chronic human wound system and evaluated both bacterial and fungal communities, thus taking into account potential inter-kingdom species interactions. Their results provided evidence to support the notion that the balance between competitive and cooperative interactions in biofilms are largely mediated by the order of species arrival. Additionally, the importance of priority effects can also be considered within the context of community coalescence (i.e., timing of arrival of entire communities). To illustrate this, Svoboda et al. (2018) experimentally evaluated the coalescence of fresh and brackish bacterioplankton communities. These authors inoculated sterile media with one of the donor communities and later the other community was inoculated at distinct time points (i.e., 0–96 h after the initial inoculation). The results revealed that the time after initial inoculation strengthens priority effects (i.e., time allowed for the establishment of the initial community), thus resulting in the lower success of taxa establishment from a later arriving community.

### Dispersal and Ecosystem Function in Agroecosystems

Microbial inoculants in agriculture are often introduced to the soil environment to promote and/or enhance specific biological functions (e.g., plant nutrient acquisition, pathogen suppression, etc.). For example, by inoculating *Pseudomonas* sp. RU47 in the soil, Nassal et al. (2018) showed an increase in phosphatase activity associated with plant growth promotion *via* improved phosphorus uptake. In another example, the inoculation of *Acinetobacter calcoaceticus* strain CSY-P13 was shown to mitigate stress from ferulic and p-hydroxybenzoic acids in cucumber by activating antioxidant enzymatic activities and altering the overall bacterial community composition (Wu et al., 2018). In addition, their results showed that the inoculation with CSY-P13 increased the activities of phosphatase, catalase, urease, and sucrase enzymes in cucumber, representing the potential for deliberate inoculations of alien microbial species to elicit changes in nutrient cycling dynamics. While shifts in local community structure and function have been widely observed in inoculation-based studies, it is yet unclear whether a single inoculation or multiple introduction events are necessary to achieve desirable goals (i.e., the importance of dispersal frequency on ecosystem function and population establishment). For example, using long-term research plots inoculated with *Bradyrhizobium japonicum*, Narożna et al. (2015) showed persistent and viable populations of this N_2_ fixing bacterium nearly 20-years after inoculation. Their results suggest that microbial inoculations with this particular species can remain viable in agroecosystems for extended periods of time, even in the absence of a suitable host. Most interestingly, in this particular case, only a single inoculation was proved to be sufficient for population establishment. In another study, however, Wang et al. (2021) examined the effects of multiple inoculation events with phosphate-solubilizing and N_2_ fixing bacteria applied individually and in combination. Their results showed the resident soil microbiome to be resilient to inoculation, indicating the potential importance of repeated attempts. Last, they found subsequent inoculations to cause a sequential impact on the local community structure. This finding aligned with their initial hypothesis that successive inoculations can result in higher persistence and potential naturalization of inoculated taxa in the system.

## Evolutionary Implications of Microbial Dispersal

Mostly due to challenges associated with properly defining the concept of species, and the fact that evolutionary changes can alter community dynamics even if new species are not created (Rainey and Travisano, 1998), the evolutionary implications of microbial dispersal can better be understood by considering how dispersal affects diversification (i.e., generation of genetic variation within populations or communities), rather than strictly focusing on speciation and species interactions. Differentiation in microbes occurs mostly by mutation and gene transfer, which in microbial systems also includes the uptake of environmental DNA (Hanson et al., 2012; Nemergut et al., 2013; Xu et al., 2020). Given their short generation times, fast growth rates, rapid genetic mutations, and gene transfer (Zhou et al., 2013, 2015; Zhou and Ning, 2017), the influence of evolutionary processes on microbial community dynamics are very important, albeit often neglected in landscape microbiome studies. These processes are–to some extent–mediated by dispersal events that determine the rate of gene flow across local and regional scales and have multifaceted eco-evolutionary implications.

### Dispersal and Microbial Gene Flow

The movement of species in space and time fundamentally relates to the movement of genetic material and gene flow (i.e., the movement of genes into or out of a population). Changes in gene flow rates are responsible for population differentiation and speciation, and can modulate the genetic structure of ecological communities (Knowles, 2009; Kisel and Timothy, 2010; Hackel and Sanmartín, 2021). Like different dispersal rates influencing patterns of species diversity in metacommunities (see above), differences in the rates of gene flow mediated by dispersal also lead to variable levels of genetic variation across populations. For example, if the rate of gene flow is too high, two populations will have equivalent genetic variation and allele frequency leading to an overall genetic homogenization. On the contrary, isolated communities/populations (i.e., under low dispersal rates) are expected to experience accelerated genetic drift and speciation from intense intraspecific differentiation (Puritz et al., 2012; Waters et al., 2020). This can result in a decrease in species genetic variation and likely enhance the susceptibility of a given population to environmental perturbation (Waters et al., 2020). As such, the consequences of differential gene flow rates can be interpreted analogously to the effects of dispersal on community diversity, functioning, and ecological properties.

Important to counterbalance the effect of genetic homogenization, microbes have different dispersal capabilities, all of which are driven by organismal intrinsic traits (motility and chemotaxis, spore formation, and dormancy) and the mechanisms of dispersal (passive and active dispersal) (see above). Some specific taxa (e.g., arbuscular mycorrhizal fungi) can reproduce and disperse *via* active and passive processes (i.e., spores, extraradical mycelium, dormancy). Such strategy reflects a broader spectrum of adaptation directly affecting the survival and genetic extension of a species (Paz et al., 2021). Besides, microbial traits linked to dispersal are also known to enhance gene flow through time scales (Jordano, 2017). For example, long-term dormancy of microbial cells can result in long-distance dispersal and/or long-term persistence, thus connecting genetically distinct communities and populations across evolutionary time scales.

### Dispersal and Genetic Rescue

In theory, the success of genetic rescue is related to three key mechanisms. First, a population previously exposed to a low level of a stressor tends to increase the population size of resistant genotypes, thus favoring rapid adaptation when similar stress or disturbances are encountered (Gomulkiewicz and Holt, 1995; Bell and Gonzalez, 2009). Second, the initial population size is often positively correlated with the probability of species adaptation and survival in face of environmental disturbances (Lanfear et al., 2014), by reducing the risk of stochastic exclusion (Gomulkiewicz and Holt, 1995; Bell and Gonzalez, 2009; Gienapp et al., 2013). And third, the connectivity of populations across a landscape (similar to the metacommunity framework) enhances gene flow and genetic variation, which promote genetic rescue (Bell and Gonzalez, 2009; Bourne et al., 2014).

**FIGURE 4:**
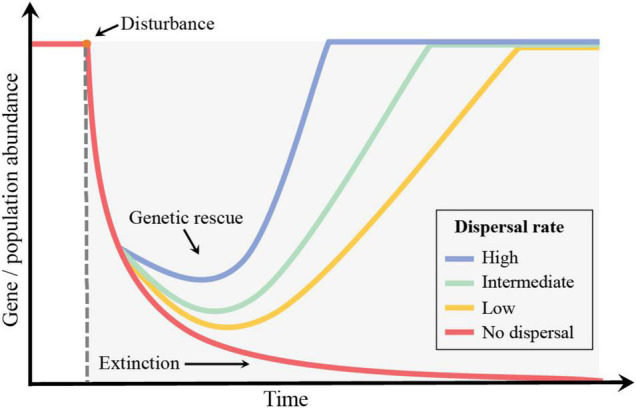
Conceptual model displaying the potential decay in gene abundance due to population decline after disturbance. Dispersal contributes to genetic rescue and avoids loss of genetic variation. Different dispersal rates relate to the pace of genetic rescue over time.

Dispersal can contributes to genetic rescue by increasing population size and consequently the rate of mutation and diversification (Figure 4) (Gomulkiewicz and Holt, 1995; Lenormand, 2002). For example, O’Connor et al. (2020) examined contrasting modes of dispersal and antibiotic selection history in *P. fluorescens* SBW25 using a gradient of antibiotic stress. This study showed that even though the previous exposure to antibiotics influenced genetic rescue in a metacommunity context, dispersal across community types and locally (i.e., dispersal through the antibiotic gradient) accelerated diversification rates. In another example, Low-Décarie et al. (2015) experimentally evaluated the importance of connectivity in soil microbial metacommunities for genetic rescue under herbicide stress. They showed that genetic rescue in a community context is dependent on the dispersal of both rare existing resistance lineages and new genotypes that arise through mutation following severe environmental degradation across the landscape.

Understanding the conditions and ecological processes that underpin the importance of dispersal affecting genetic rescue has implications for conservation biology and for the management of resistant pathogens (Alexander et al., 2014). For example, Cheptou et al. (2017) showed that highly fragmented systems result in negative effects on population rescue, even when sufficient adaptation is present, by driving the species to extinction and endangering the metapopulation dynamics. Furthermore, the beneficial association of microbes with hosts has also been shown to enhance host species rescue by increasing host fitness and enhancing survival chances (Mueller et al., 2020). In the case of pathogens, it was argued that globalization greatly contributed to the increased rate of evolution in resistant strains directly affecting their dispersal potential (Baquero et al., 2021). Several studies have shown the emergence of microbial resistant strains to pesticides and antibiotics, collectively highlighting an increased risk for agricultural systems and human health (Thanner et al., 2016; Hudson et al., 2017; Hernando-Amado et al., 2020). For example, antibiotic resistant microbes and their infections have been shown to affect access to healthcare and incur high costs of treatment (Hernando-Amado et al., 2020). In the United States alone, additional costs associated antibiotic resistance are estimated to increase $30 billion annually (van Duin and Paterson, 2016). Ecological studies can promote further understanding of microbial resistance pathways and strategies to improve control of antibiotic resistance. For instance, metapopulation theory can be used to understand emergence of resistant pathogens by incorporating dispersal barriers and population rescue decline (Koch et al., 2017).

Worth mentioning and different from macro-organisms, microbes are capable of uptaking genetic materials from the environment (i.e., dead cells or environmental DNA fragments). This illustrates a type of genetic rescue unique to microbes that is associated with the persistence and maintenance of genetic variation (or genes) rather than population rescue. For example, following exposure to treated wastewater containing free extracellular antibiotic resistance genes (eARGs), *Staphylococcus aureus* strains were found to be newly resistance to methicillin (Naquin et al., 2015). This study showed a successful natural extracellular transformation during chemical stress and provided evidence of resistance gene assimilation (most likely *mec*A—a methicillin resistance gene). In addition, Olwal et al. (2018) showed an increase in eDNA release by *S. epidermidis* biofilms in response to sub-lethal heat and oxidative stress. As such, the authors suggested that eDNA release could act as a potential mechanism underlying species resistance under physicochemical stress. Overall, this implies that the ability to incorporate extracellular eDNA can improve species survival and boost genetic rescue by upregulating stress resistant genes in stressful environments.

### Dispersal in an Eco-Evolutionary Context

Dispersal is directly associated with the ecology and evolution of species interactions. A classic example comes from one of the most widespread and ecologically important instances of mutualism: the association of terrestrial plants with fungal mycorrhizal symbionts. This association allowed early land plants to undergo the ecological transition to terrestrial life (Delwiche and Cooper, 2015; Morris et al., 2018), leading to both the dispersal and evolutionary radiation of the mycorrhizal fungi and terrestrial plants. Such examples of assisted dispersal by beneficial interaction occur more often than expected. Ingham et al. (2011) experimentally demonstrated beneficial co-dispersal between the non-mobile fungus *Aspergillus fumigatus* and the swarming bacterium *Paenibacillus vortex.* They showed *P. vortex* to rescue *A. fumigatus* from inhospitable environments and the transport of *A. fumigatus* conidia to areas containing a relatively higher concentration of antibiotics, which creates an environment more favorable to the fungal germination and lower bacterial competition. Furthermore, the authors hypothesized that *A. fumigatus* hyphae can facilitate bacterial movement through an otherwise impassable heterogeneous soil matrix, thus resulting in a relationship beneficial to both species. Other studies have provided support for this hypothesis by showing that the fungal mycelia can act as dispersal channels (or “fungal highways”), facilitating bacterial dispersal even under unfavorable conditions and environmental heterogeneity (Kohlmeier et al., 2005; Wick et al., 2007). To illustrate the concept, the hyphae of *Phomopsis liquidambaris* was shown to assist the migration of rhizobia from bulk soil to the rhizosphere of peanut (*Arachis hypogaea*) (Zhang et al., 2020). In another eco-evolutionary perspective, these fungal highways were investigated with respect to the movement of species associated with predator-prey interactions. Otto et al. (2017) experimentally evaluated the ability of the gram-negative bacterial predator *Bdellovibrio bacteriovorus* to utilize fungal mycelia as dispersal channels to locate suitable prey, in this case, *Pseudomonas fluorescens*. Although they found no predator dispersal through the fungal mycelia in the absence of prey, the predatory bacterium utilized the fungal mycelial highway when the prey was available. Last, these authors suggested that dispersal of predators *via* fungal hyphae is “activated” by the presence of the prey serving as a source of carbon and energy supply.

Dispersal can also mediate the evolutionary dynamics of competition-colonization trade-offs (Urban et al., 2020). Dispersal traits are expected to evolve as a consequence of species escaping competition or evading disturbance events, thus enhancing species survival and persistence at regional scales (Duputié and Massol, 2013). Conversely, it could be expected that the benefit of dispersal abilities decreases as species locally adapt and face an increased cost of dispersal, such as in the case with increased landscape fragmentation (Cenzer and M’Gonigle, 2019). To illustrate this, Dini-Andreote et al. (2018) investigated microbial adaptative traits along a gradient of saltmarsh formation. These authors revealed an overrepresentation of dispersal-related traits at a community level (i.e., chemotaxis and flagellar motility) in earlier stages of land formation, reflecting community dynamics in environments with frequent environmental change (i.e., flooding events). On the other hand, the gradual transition to a less diffusible habitat (i.e., the soil matrix) reflected in a progressive increase in the abundance of ARGs (i.e., competitive traits), in addition to a more versatile metabolism of carbohydrate-active enzymes. In sum, this study links a set of specific microbial traits directly associated with dispersal-competition trade-off during the eco-evolutionary transition of land colonization.

## Concluding Remarks

Dispersal has long been recognized as one of the fundamental processes structuring ecological communities. Foundational studies have demonstrated its importance for species movement and how dispersal interacts with other assembly processes, such as selection (Ron et al., 2018). However, its importance in microbial systems has–to some extent–been neglected, mostly due to technical limitations, but also given the seminal paradigm of “everything is everywhere.” Together, these factors have accounted for our yet inability to properly appreciate the importance of dispersal in structuring microbial communities; albeit seminal work has been done in explicitly studying dispersal in microbial systems. On the other hand, while large-scale experimental manipulations focusing on dispersal in macro-organismal systems remain unrealistic, the nature of microbial systems provides an easily manipulatable system, apt for experimentation that can undoubtedly further our understanding of dispersal on both the micro- and macro-ecological scales. With this, we argue that the field of ecology will benefit from future research that considers dispersal and its influence on taxonomic and functional diversity, as well as the influence of historical contingencies and community coalescence in microbial systems. Additionally, the importance of dispersal affecting eco-evolutionary outcomes deserves renewed attention due to expected increases in exotic invasion and gene flow across systems as a result of intensifying climate change and globalization.

## Author Contributions

GC, LB, and FD-A wrote, discussed, and reviewed the entire manuscript. All authors contributed to the article and approved the submitted version.

## Conflict of Interest

The authors declare that the research was conducted in the absence of any commercial or financial relationships that could be construed as a potential conflict of interest.

## Publisher’s Note

All claims expressed in this article are solely those of the authors and do not necessarily represent those of their affiliated organizations, or those of the publisher, the editors and the reviewers. Any product that may be evaluated in this article, or claim that may be made by its manufacturer, is not guaranteed or endorsed by the publisher.
